# Dr. Thompson's Reply to the Remarks of Mr. Fogo

**Published:** 1812-10

**Authors:** D. Thompson

**Affiliations:** Whitby


*To the Editors of the Medical and Physical Journal.*

GENTLEMEN,

IN the 162d Number of your Journal, you did me the favor to insert a case of diseased liver, the history and treatment of which, as far as I was acquainted with them, I endeavored to relate with fidelity, and, I hope, with sufficient modesty. If I hazarded any physiological opinions, I offered them merely as conjectures, and considered them very subordinate to the plain practical statement of facts which preceded them; nor am I aware that my paper contained one illiberal remark, or uncandid insinuation. I was, therefore, not a little surprised to find it commented upon by Mr. Fogo, of Newcastle, in a tone very little consonant to philosophical investigation, or professional politeness; and, much as I dislike even literary altercation, and little as I deem Mr. Fogo's comments, viewed as a piece of criticism, deserving of notice, I must yet trouble you with a few remarks in reply.

If I were to notice the whole of Mr. Fogo's performance, I should have not only to advocate my own cause, but also to defend the reputation of others infinitely above me; for I find the respected names of Cullen, Darwin, Rotherham, and Gregory, treated with as little ceremony as my own. The great, the little, professors, and private practitioners, are are included in one sweeping accusation of ignorance; and, while I confess to Mr. Fogo that I feel flattered by being placed amid such distinguished company, I can assure him, that nothing but a regard for their reputation, and my own, prevents my introducing him also among the number.

Mr. Fogo, after having given an abridged quotation of my statement of symptoms, rendered a little uncouth by the pruning which he has bestowed upon it, declares with the triumphant air of a man who has made an important discovery, that the disease in question was "a case of genuine chronic hepatitis," meaning, of course, when it came under my notice, as his intention is evidently to censure me, because "I did not choose to give it a name." I am aware of the little value of mere verbal criticism, yet, in the hope of lowering Mr. Fogo's self-sufficiency, I shall venture to call the accuracy of his judgment in question. Hepatitis, agreeable both to the etymology of the word, and the definitions of all nosologists, implies merely an inflammation of the liver, and cannot, I presume, be with propriety applied to the suppurative state of that viscus, which is the result of inflammation, and which was precisely its state in my patient. I described the symptoms, and pointed out the seat and nature of the complaint with sufficient accuracy; and, had I conceived it necessary to put its name at the head of my paper, I should certainly have called it an abscess, or apostema, of the liver; and this I believe would have been strictly proper: whereas Mr. Fogo, by pronouncing it, in that state, chronic hepatitis, has completely confounded cause and effect; as well might he call an empyema a pleurisy.

Mr. Fogo next remarks, that I neglected to state whether the patient had taken any medical advice, or medicine, previous to his application to me. I did neglect that statement, or rather I purposely omitted it, and for reasons which, I fear, will never influence him—a desire to avoid even the appearance of ostentation, or of having insinuated any thing to the prejudice of my professional neighbours. But Mr. Fogo asserts, that whether the patient applied previously for advice or not, is of little consequence, "as he would have got no medicines necessary to remove the obstruction of the biliary, or other, (what other?) vessels in the liver, in the early stage of the disease." I am really astonished that any person laying claim to the character of a gentleman, should have dared to cast so illiberal a reflection upon the whole medical department of this neighbourhood. By what right, or upon what authority, does Mr. Fogo presume to pronounce us all thus ignorant? Does he know any thing of our our practice, of our education, of our intellect? or does he only measure our professional abilities by his own? I am unwilling to make use of coarse language, or I should reply to this remark in terms which, however much they might disgrace me, would, I think, be not unaptly applied to him.

After this specimen of the politeness of his sentiments, Mr. Fogo next furnishes us with one of the accuracy of his style. "Dr. Thompson (he observes) may perhaps be surprised at the *name* of chronic hepatitis; for, if he has acquired no more knowledge of it than he received from the lectures and writings of our late venerable professor, he will know very little about it, except its *name!*" The lectures and writings of our late venerable professor might, at least, I should have thought, have taught him how to write four lines without a contradiction. I may well be surprised at the name of a disease which Dr. Cullen knew very little about, particularly when I find that he both lectured and wrote upon it, and that this very disease, owing to the many persons who return thither affected with it from tropical climates, is perhaps more frequently met with in Edinburgh, than in any other place of equal extent in Britain. The rude and illiberal insinuation intended to be conveyed under the above remark, is beneath my notice.

The next section of Mr. Fogo's paper is wholly taken up with exposing the stupidity of Doctors Cullen and Rotherham; and in pointing out the "fatal blindness, ignorance, and *security*, of the profession in general." If we be thus blind, it is indeed fortunate for us that we are secure, or we might, peradventure, all fall into a ditch together: and, as to the attainment of learning, our eyes, it seems, could be of little use to us, as Mr. Fogo assures us "that there is no information to be got from any book, either ancient or modern."

I am next accused of having repeated Dr. Cullen's sentiments, and, as the charge is no crime, I shall not attempt to refute it; though I was really unconscious of having on my side such respectable authority. I have ventured also, it appears, to wish for a more systematic arrangement of the *diseases* of the liver, and of the *symptoms* by which they may be known and distinguished; and this desideratum Mr. Fogo attempts to supply, by informing me that "the *anatomical structure* of the liver is as well known as that of any other gland." He tells me also, that this knowledge "is quite sufficient for practical purposes," though "our knowledge of the manner in which secretion is performed in the various glands is very contracted," and though "the knowledge of the nature and functions of that very important organ the liver, which the profession is in possession of, is very small;" and all this he tells me, *currente calamo*, without once pausing to breathe, or to consider what he is uttering.

I am next represented as vacillating between two opinions; because, while I acknowledge the importance of those functions well known to be performed by the liver, I suspect it may also perform others equally or more important, though less clearly understood; and, from this supposed vacillation of my opinion of the hepatic functions, Mr. Fogo tells me, with his usual politeness, that he doubts whether I should have known how to treat my patient's case, had I seen him sooner. This remark clearly shows the disposition of its author. Too ungenerous to praise, and finding nothing to censure, he relinquishes facts, and continues his attack with doubts and surmises.

Mr. Fogo next asserts, with his accustomed perspicuity, that my "comparing the functions of the liver to those of the kidney, is a degradation." Degradation! To what? To whom? To the dignity of the liver? To his understanding, or to my own? If he means by this vague remark, that the functions of the liver are more essential to the animal economy, or that their derangements produce more danger or distress than those of the kidneys, I am compelled to tell him he is incorrect. Let him observe the degrees of suffering produced by hepatitis and nephritis; let him compare the effects of ischuria renalis, with those of an obstruction of the bile, and he will soon alter his opinion.

There is little in the remaining part of Mr. Fogo's paper that immediately concerns myself; but he has no sooner quitted me, than, unmindful of that excellent maxim, *de mortuis, &c.* he falls with equal malevolence upon the character of Dr. Paxton. I was compelled to mention that gentleman's name, and I hope I did it with delicacy and respect; but Mr. Fogo spares neither the living nor the dead; he comments with unfeeling severity upon the errors of those who can err no more, and, in his progress of detraction, invades even the silent sanctuary of the tomb. The unhandsome insinuations which he has thrown out against myself, merit my contempt. I feel very little necessity for an appeal to his judgment or erudition; and, had not his illiberality been more conspicuous than either, I should not have noticed him at all. His essay, viewed as a piece of criticism, is utterly unworthy attention; for, though he objects to some of my opinions, he has refuted none; but has merely opposed to them those common-place doctrines, which I never called in question, and which every apothecary's apprentice could have repeated as well as himself. He has entirely entirely disregarded the reasons which led me to suspect that the hepatic system might possibly effect more important purposes than the mere secretion of bile; he even seems ignorant that any other functions were ever ascribed to it; from which I infer, that his acquaintance with physiology and physiological writings, is exceedingly small; and, from his having failed to detect an anatomical error, which, I know not how, crept into my last paper, and which, had he detected it, I have no doubt he would gladly have exposed, I am induced to believe that his knowledge of the structure and situation of parts is equally limited. The error I allude to, is where an adhesion is stated to have taken place between the *upper* surface of the liver and the stomach, and where the words *under*, or *concave*, surface, should undoubtedly have been used.

Considered as a practical essay, Mr. Fogo's paper is of little value; for, though he more than insinuates that he is the only person capable of detecting and curing chronic hepatitis, yet he gives us no one proof that he actually does possess even this very moderate degree of information. He says, indeed, very wisely, that, "if we find that any gland is not secreting its proper fluid in *sufficient* quantity, we must consider the gland as in a diseased state, and endeavor to restore it to its healthy state." I presume that a secretion may be morbidly increased, as well as morbidly decreased; I also think that it may undergo other changes besides those of quantity, though I believe they are generally united; and therefore it appears, that Mr. Fogo has taken a very contracted view of the subject; he has intirely overlooked one state of disease, and has given us no rules for discovering or removing the other. I need no instruction from Mr. Fogo upon the uses of the bile, but I am far from being satisfied that any changes in it can produce those numerous, irregular, and destroying, symptoms, vaguely termed bilious. The cause does not appear adequate to the effect; and, therefore, for this and other reasons, which I need not recapitulate, I doubt if we yet fully understand the nature and *extent* of the hepatic functions. It was in the hope of arousing the attention of the profession to this interesting and difficult subject, that I took the liberty of throwing out the hints contained in my last paper, and I shall be much gratified to see it taken up by some person capable of doing it justice.

I am, your's, &c.

D. THOMPSON, M.D.

*Whitby, Aug.* 10, 1812.

